# Overall and disease-free survival in patients with HPV-positive and HPV-negative oropharyngeal cancer

**DOI:** 10.31744/einstein_journal/2025AO1525

**Published:** 2025-08-15

**Authors:** Matheus de Abreu, Dandara Menezes de Araujo Oliveira, Bartolomeu Conceição Bastos, Janaina Naiara Germano, Luiz Paulo Kowalski, Maria Paula Curado

**Affiliations:** 1 A. C. Camargo Cancer Center International Research Center Epidemiology and Statistics Nucleus São Paulo SP Brazil Epidemiology and Statistics Nucleus, International Research Center, A. C. Camargo Cancer Center, São Paulo, SP, Brazil.; 2 A. C. Camargo Cancer Center Department of Head and Neck Surgery and Otorhinolaryngology São Paulo SP Brazil Department of Head and Neck Surgery and Otorhinolaryngology, A. C. Camargo Cancer Center, São Paulo, SP, Brazil.

**Keywords:** Oropharyngeal neoplasms, Squamous cell carcinoma of head and neck, Human papillomavirus viruses, Recurrence, Survival rate, Disease-free survival, Prognosis

## Abstract

**Objective:**

To analyze the overall and disease-free survival of patients with oropharyngeal squamous cell carcinoma.

**Methods:**

Sociodemographic and clinical data, HPV status, and alcohol/tobacco consumption were assessed among patients treated at *AC Camargo Cancer Center*, São Paulo, Brazil. Absolute and relative frequencies, overall survival, and disease-free survival at 5 and 7 years were calculated using Kaplan-Meier analysis, and Cox regression was used to estimate the risk of death.

**Results:**

The 7-year overall survival rate was 61.6% for patients with oropharyngeal squamous cell carcinoma (67.5% for HPV-positive cases and 51.1% for HPV-negative cases). Among HPV-positive cases, the risk of death was 5.29-fold higher for smokers than that for non-smokers (p<0.001) and 4.42-fold higher for patients with metastasis than that for those without (p=0.108). Among HPV-cases, those with clinical stage T3/T4 disease had a 1.8-fold higher risk of death than those with T1/T2 disease (p=0.039), and those with metastasis exhibited a 4.62-fold higher risk than those without (p<0.001). The 7-year disease-free survival rate for oropharyngeal squamous cell carcinoma was 88.6% (90.1% for HPV-positive cases and 85.9% for HPV-negative cases). Among HPV-positive cases, patients with metastasis had an 11.75-fold higher risk of recurrence than those without (p<0.001); among HPV-negative cases, patients with metastasis had a 4.62-fold higher risk than those without (p=0.004).

**Conclusion:**

Patients with HPV-positive oropharyngeal squamous cell carcinoma had higher overall survival and increased recurrence rates within the first 3 years but lower recurrence rates beyond 5 years than patients with HPV-negative disease. More frequent follow-ups after 5 years are recommended for patients with HPV-positive disease. Tobacco and alcohol consumption negatively affect the overall survival of patients with HPV-positive oropharyngeal squamous cell carcinoma.

## In Brief

This retrospective cohort study highlights the differences in survival and recurrence patterns between patients with HPV+ and those with HPV- oropharyngeal cancer. Patients with HPV+ disease had better overall survival but early recurrence, while those with HPV- disease exhibited later recurrence. Smoking, alcohol consumption, and metastasis worsened the outcomes, emphasizing the need for continuous follow-up.

## Highlights

■ HPV+ is associated with improved overall survival in oropharyngeal cancer
■ HPV+ tumors tend to recur earlier than HPV- tumors.
■ Metastasis is a key prognostic factor for mortality and recurrence regardless of HPV status.
■ Smoking and alcohol consumption reduce the favorable prognosis of HPV+.

## Graphical abstract

**Figure:**
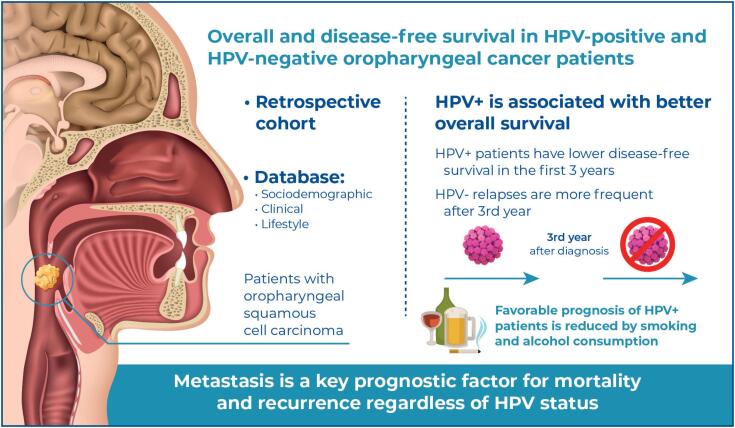

## INTRODUCTION

Oropharyngeal squamous cell carcinoma (OPSCC) affects the tonsils, base of the tongue, soft palate, and uvula.^(1)^ In 2022, an estimated 106,400 new OPSCC cases and 52,305 related deaths occurred worldwide.^(2)^ Oropharyngeal squamous cell carcinoma is associated with alcohol and tobacco use,^(3)^ as well as human papillomavirus 16 (HPV) infection,^(4,5)^ which is present in approximately 30% of new OPSCC cases.^(6,7)^

The incidence of HPV-associated tumors has increased in recent years.^(8)^ According to one estimation, the majority of head and neck cancers in England will be HPV-positive (HPV+) within 20 years.^(9)^ In developed countries, HPV-attributable OPSCCs represent 17% to 56% of cases, while in less developed countries, the incidence is 13%.^(10,11)^ In Brazil, the prevalence of HPV+ OPSCC ranges from 4.0% to 59.1%.^(12)^ The sociodemographic profile of patients with HPV+ OPSCC is typically younger, male, white, and non-smoking.^(13-15)^ The most common sites for HPV+ tumors are the tonsils and base of the tongue, and such tumors are often associated with cervical lymph node disease.^(16)^

HPV-negative (HPV-) OPSCC has lower overall survival (OS) and disease-free survival (DFS) rates; the reported 3-year OS is 82% for patients with HPV+ disease compared with only 45% for those with HPV- disease.^(17)^ Thus, the HPV infection status in the tumor tissue is a prognostic factor.^(18)^ The combination of an HPV infection with other risk factors, such as alcohol and tobacco use, can negatively affect survival rates.^(18,19)^ Certain studies have indicated that factors such as the anatomical site and histological subtype also influence a patient's prognosis and survival. For instance, malignancies in the lymphoepithelial regions of the oropharynx, such as the tonsils and base of the tongue, have more favorable prognoses than those in non-lymphoepithelial subsites.^(20-22)^

HPV+ tumors have demonstrated a better response to treatment and higher survival rates than HPV- tumors.^(23-25)^ The reported risk of death from HPV+ OPSCC is 3.4 times lower than that from HPV- OPSCC.^(26)^ The two subtypes also have different patterns of late recurrence-.^(27,28)^

## OBJECTIVE

To analyze and compare the 5- and 7-year overall survival and disease-free survival rates of patients treated for HPV+ and HPV- oropharyngeal squamous cell carcinoma at a Brazilian cancer center and to identify associated prognostic factors.

## METHODS

### Sample population

For this retrospective cohort study, we included patients with data available from the Hospital Cancer Registry of *A.C. Camargo Cancer Center* (ACCCC), São Paulo, Brazil for the period 2000-2022. All the patients had a primary diagnosis of squamous cell carcinoma (MORFO 8070/3) of the oropharynx. Cases were classified as follows, according to the third edition of the International Classification of Diseases for Oncology codes: C01.9 (base of tongue), C05.1 (soft palate), C05.2 (uvula), C09.0 (tonsil, tonsillar fossa), C09.1 (tonsil, tonsillar pillar), C09.8 (overlapping lesion of the tonsil), C09.9 (tonsil, not otherwise specified [NOS]), C10.0 (vallecula), C10.1 (anterior surface of epiglottis), C10.2 (lateral wall of oropharynx), C10.3 (posterior wall of oropharynx), C10.8 (overlapping lesion of oropharynx), and C10.9 (oropharynx, NOS).

Sociodemographic information collected included sex (male and female), age group (18-39, 40-49, 50-69, and 70+ years), self-declared race (white, black, brown, and yellow), and educational level (0-8, 8-11, and >11 years). Lifestyle variables included smoking and alcohol consumption (yes, no, and former). Cases were staged according to the eighth edition of the American Joint Committee on Cancer manual. Clinical information included the presence of a second primary tumor at diagnosis or follow-up, HPV infection status determined via p16 immunohistochemistry (IHC), disease recurrence, year of diagnosis, and last status (alive with cancer, alive NOS, death due to cancer, and death NOS). The follow-up times for survival analysis were the 1^st^, 3^rd^, 5^th^, and 7^th^ years.

This study was approved by the Human Research Ethics Committee of *A. C. Camargo Cancer Center* (CAAE: 80177317.9.0000.5432; #7.234.641) and was conducted in compliance with the tenets of the World Medical Association Declaration of Helsinki.

### Immunohistochemistry for p16

Samples were tested for p16 expression by using IHC. IHC was performed on 4-μm sections of paraffin-embedded tumor blocks by using a monoclonal mouse anti-human p16INK4a antibody, E6H4TM clone (Ventana Medical Systems), and the OptiView DAB IHC Detection Kit (Ventana Medical Systems) on a BenchMark ULTRA system (Ventana Medical Systems), according to the manufacturer's specifications. Cases with positive p16 expression in more than 70% of the tumor cells were classified as HPV+ OPSCC. Analyses were performed by an operator who was blinded to the patients’ clinical information.

### Statistical analysis

Descriptive statistics (absolute and relative frequencies) were used to present the sociodemographic and clinical characteristics of the patients. The χ^2^ test was used to compare categorical variables, with p<0.05 considered significant. Survival rates were stratified according to HPV infection for the 1^st^, 3^rd^, 5^th^, and 7^th^ years. The OS rate was determined using the date of diagnosis and the patient's vital status (alive or deceased) at the last follow-up or through active searches on official platforms. DFS was determined using the dates of primary tumor diagnosis and tumor recurrence. The Kaplan-Meier method was used to calculate survival rates by the 1^st^, 3^rd^, 5^th^, and 7^th^ years, and the log-rank test was used to compare survival curves. Univariate (Tables 1S to -4S, Supplementary Material) and multiple Cox regression analyses were used to estimate the hazard ratio (HR) and its corresponding 95% confidence interval (95%CI). For multiple regression analysis, variables with p<0.20 in the univariate analyses were selected. The stepwise method was used, adding variables to the model according to their p-values, from the smallest to the largest. The final model was built by adding variables according to the following premises: 1. no change in HR >10%; 2. improved precision (narrower 95%CI); 3. total degrees of freedom allowed for the outcomes; and 4. interaction effects between covariates. The Schoenfeld residual scale was used to test the proportional risk conferred by each variable. All statistical analyses were performed using IBM SPSS Statistics for Windows, Version 25.0 (IBM Corp., Armonk, NY, USA).

## RESULTS

Of the 448 included patients, 292 had HPV+ OPSCC (65.2%) and 156 had HPV- OPSCC (34.8%). Among them, 79.5% (n=232) and 84.6% (n=132) were male, respectively. The median (range) age was 61 (37-88) years for patients with HPV+ disease and 63 (36-96) years for those with HPV-disease. The majority of patients were aged 50-69 years in both groups. White individuals accounted for 80.3% of HPV+ cases and 72.7% of HPV- cases. The most frequently affected site in HPV+ cases was the tonsils (47.9%, n=140), whereas that in HPV- cases was the base of the tongue (28.2%, n=44). The clinical stage was IV in 52.9% of HPV- cases and 12.5% of HPV+ cases. Lymph node involvement (N+) was present in 82.0% of HPV+ cases (n=232) and 61.9% of HPV- cases (n=91). Recurrence was detected in 7.5% of HPV+ cases and 11.5% of HPV- cases. Among patients with OPSCC HPV+, 44.6% (n=128) were non-smokers and 44.1% (n=123) were non-drinkers; in contrast, among those with HPV- OPSCC, 48.7% (n=76) were smokers and 48.4% (n=75) were drinkers (Table 1).

**Table 1:** Sociodemographic and clinical characteristics of patients with HPV+ and HPV- oropharyngeal squamous cell carcinoma

Variable		Total (%) n=448	HPV+ (%) n=292	HPV- (%) n=156
Sex				
	Male	364 (81.3)	232 (79.5)	132 (84.6)
	Female	84 (18.8)	60 (20.5)	24 (15.4)
Age		-		
	18-49 years	49 (10.9)	37 (12.7)	12 (7.7)
	50-69 years	294 (65.6)	193 (66.1)	101 (64.7)
	70+ years	105 (23.4)	62 (21.2)	43 (27.6)
Self-declared race/ethnicity				
	White	308 (77.8)	212 (80.3)	96 (72.7)
	Black	21 (5.3)	9 (3.4)	12 (9.1)
	Brown	61 (15.4)	37 (14.0)	24 (18.2)
	Yellow	6 (1.5)	6 (2.3)	0 (0,0)
	No information	52	28	24
Education				
	0-8 years	51 (22.5)	18 (11.8)	33 (44.0)
	9-11 years	50 (22.0)	37 (24.3)	13 (17.3)
	> 11 years	126 (55.5)	97 (63.8)	29 (38.7)
	No information	221	140	81
Year of diagnosis				
	2000-2010	30 (6.7)	15 (5.1)	15 (9.6)
	2011-2016	175 (39.1)	111 (38.0)	64 (41.0)
	2017-2022	243 (54.2)	166 (56.8)	77 (49.4)
Anatomical region				
	Base of tongue	121 (27.0)	77 (26.4)	44 (28.2)
	Soft palate	37 (8.3)	7 (2.4)	30 (19.2)
	Tonsils	183 (40.8)	140 (47.9)	43 (27.6)
	Oropharynx	107 (23.9)	68 (23.3)	39 (25.0)
Clinical stage				
	*In situ*	4 (0.9)	0 (0,0)	4 (2.6)
	I	63 (14.3)	50 (17.4)	13 (8.5)
	II	123 (27.9)	107 (37.2)	16 (10.5)
	III	134 (30.4)	95 (33.0)	39 (25.5)
	IV	117 (26.5)	36 (12.5)	81 (52.9)
	No information	7	4	3
TNM classification (T)				
	Tis	4 (0.9)	0 (0.0)	4 (2.7)
	T1/T2	204 (47.9)	149 (53.6)	55 (37.2)
	T3/T4	218 (51.2)	129 (46.4)	89 (60.1)
	No information	22	14	8
TNM classification (N)				
	N0	107 (24.9)	51 (18.0)	56 (38.1)
	N+	323 (75.1)	232 (82.0)	91 (61.9)
	No information	18	9	9
TNM classification (M)				
	Yes	39 (8.9)	23 (8.0)	16 (10.7)
	No	398 (91.1)	264 (92.0)	134 (89.3)
	No information	11	5	6
Recurrence				
	Yes	40 (8.9)	22 (7.5)	18 (11.5)
	No	408 (91.1)	270 (92.5)	138 (88.5)
Smoking status				
	Smoker	141 (31.8)	65 (22.6)	76 (48.7)
	Former smoker	150 (33.9)	94 (32.8)	56 (35.9)
	Non-smoker	152 (34.3)	128 (44.6)	24 (15.4)
	No information	5	5	0
Alcohol consumption				
	Drinker	201 (46.3)	126 (45.2)	75 (48.4)
	Former drinker	83 (19.1)	30 (10.8)	53 (34.2)
	Non-drinker	150 (34.6)	123 (44.1)	27 (17.4)
	No information	14	13	1

**Figure 1:**
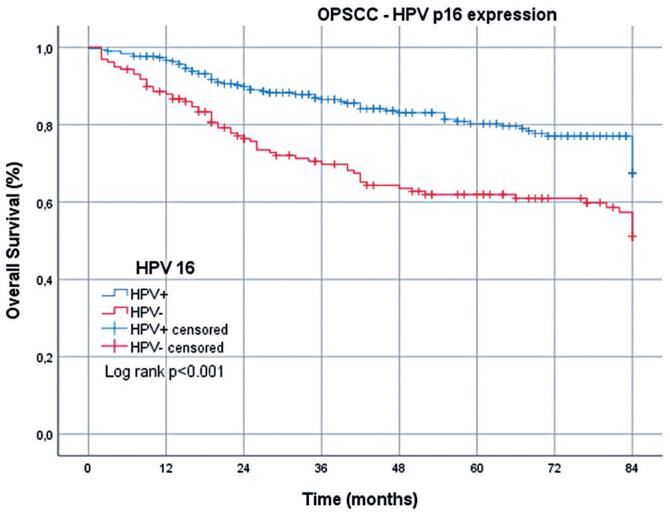
Overall survival of patients with HPV+ and HPV- oropharyngeal squamous cell carcinoma over 7 years

The mean OS for patients with OPSCC was 67.9 (95%CI=65.2-70.5) months: 72.3 (95%CI=69.4-75.2) months for HPV+ cases and 59.8 (95%CI=54.7-65.0) months for HPV- cases. By the 7^th^ year, the survival rate among patients with HPV+ OPSCC was 67.5%, compared with 51.1% among those with HPV- OPSCC, a significant difference (Table 2 and Figure 1).

**Table 2:** Overall survival rate (%) in patients with oropharyngeal squamous cell carcinoma at 1, 3, 5, and 7 years, stratified according to p16/HPV immunohistochemistry

		Time after diagnosis			
All deaths		1 year	3 years	5 years	7 years
	HPV+	96.6	86.4	80.2	67.5
	HPV-	87.8	69.7	61.9	51.1
Death from cancer		1 year	3 years	5 years	7 years
	HPV+	97.9	90.6	88.0	82.6
	HPV-	90.9	78.9	71.7	67.3
Death from other causes		1 year	3 years	5 years	7 years
	HPV+	98.6	95.3	91.2	82.0
	HPV-	96.7	88.4	86.3	76.1

**Figure 2:**
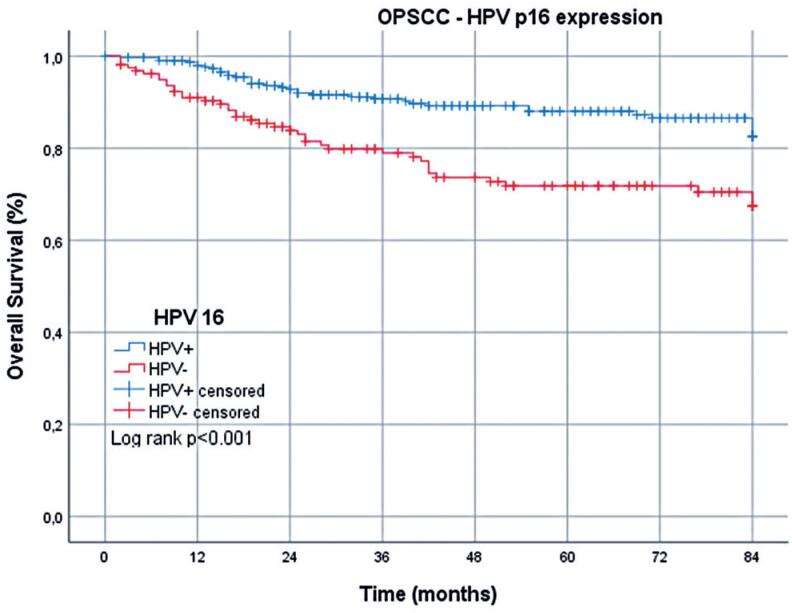
Overall survival of patients with HPV+ and HPV- oropharyngeal squamous cell carcinoma over 7 years: cancer-specific deaths

**Figure 3:**
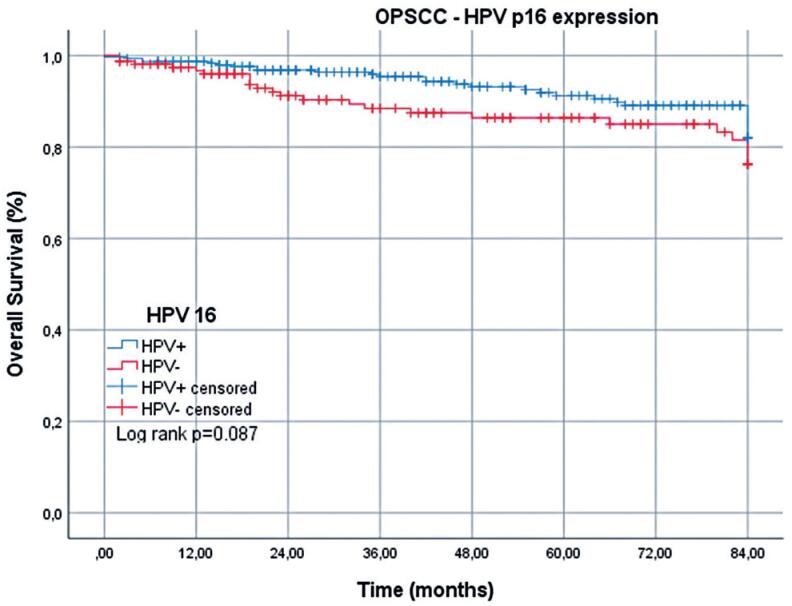
Overall survival of patients with HPV+ and HPV- oropharyngeal squamous cell carcinoma over 7 years: non-cancer-related deaths

Regarding cancer-specific deaths, the mean OS for patients with OPSCC was 73.1 (95%CI=70.7-75.4) months: 76.6 (95%CI=74.1-79.1) months for HPV+ cases and 66.5 (95%CI=61.7-71.4) months for HPV- cases. By the 7^th^ year, the survival rate among patients with HPV+ OPSCC was 82.6%, compared with 67.3% among those with HPV- OPSCC, a significant difference (Table 2 and Figure 2). Regarding non-cancer-related deaths, the mean OS for patients with OPSCC was 77.8 (95%CI=75.9-79.6) months: 79.1 (95%CI=77.1-81.1) months for HPV+ cases and 75.1 (95%CI=71.3-79.0) months for HPV- cases. By the 7^th^ year, the survival rate among patients with HPV+ OPSCC was 82.0%, compared with 76.1% among those with HPV- OPSCC (Table 2 and Figure 3).

In the multiple Cox regression analysis for patients with HPV+ OPSCC, smokers had a 5.29-fold higher risk of death than nonsmokers, whereas former smokers had a 3.2-fold higher risk. In that same group, patients with N+ tumors had a 49% lower risk of death than those with N0 tumors, whereas those with distant metastases had a 4.42-fold higher risk of death than those without (Table 3). Patients with HPV- OPSCC patients with T3 or T4 tumors had an 83% higher risk of death than those with T1 or T2 tumors, and those with metastatic disease had a 4.62-fold greater risk of death than those without (Table 3).

**Table 3:** Multiple Cox regression model for mortality risk in patients with HPV+ and HPV- oropharyngeal squamous cell carcinoma

Variable		HR	HPV+				HPV-		
			95% CI		p value	HR	95% CI		p value
			Lower	Upper			Lower	Upper	
Clinical stage									
	I/II	1.00	-	-	-				
	III	2.47	1.36	4.51	0.003*				
	IV	1.56	0.24	10.19	0.640				
TNM classification (T)									
	T1/T2					1.00	-	-	-
	T3/T4					1.83	1.03	3.26	0.039*
TNM classification (N)									
	N0	1.00	-	-	-				
	N+	0.51	0.28	0.94	0.03*				
TNM classification (M)									
	No	1.00	-	-	-	1.00	-	-	-
	Yes	4.42	0.72	27.00	0.108	4.62	2.38	8.98	<0.001*
Smoking status									
	Non-smoker	1.00	-	-	-				
	Smoker	5.29	2.25	12.44	<0.001*				
	Former smoker	3.25	1.40	7.51	0.006*				
Alcohol consumption									
	Non-drinker	1.00	-	-	-				
	Drinker	0.58	0.28	1.19	0.140				
	Former drinker	1.21	0.52	2.78	0.649				

* Statistically significant (p<0.05).

The educational level was not included in the multivariable analysis because of the high number of missing values (missing n=221, 49.3%).

**Figure 4:**
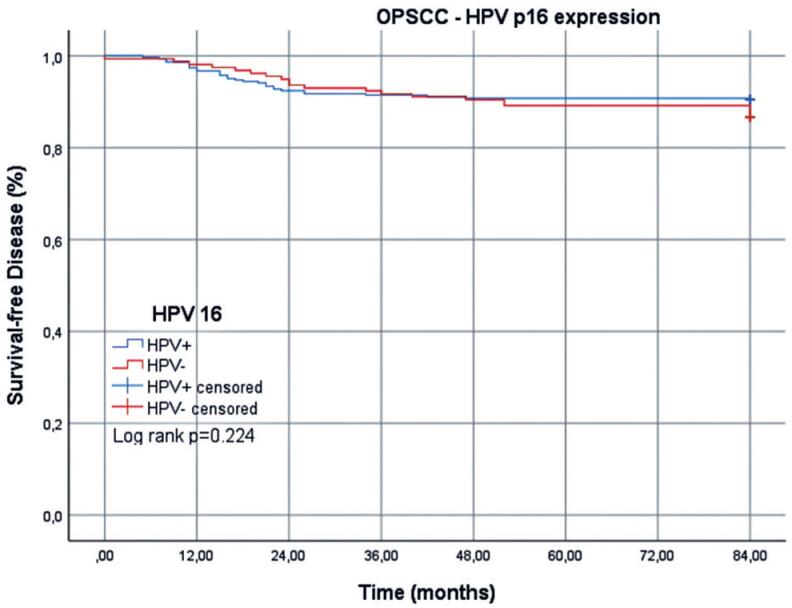
Disease-free survival of patients with HPV+ and HPV- oropharyngeal squamous cell carcinoma over 7 years

The mean DFS for patients with OPSCC was 77.6 (95%CI=75.8-79.4) months: 77.7 (95%CI=75.4-79.9) months for HPV+ cases and 77.5 (95%CI=74.5-80.5) months for HPV- cases (Table 4 and Figure 4). In the Cox multiple regression analysis, patients with HPV+ OPSCC with distant metastasis had a risk of recurrence 11.75-fold higher than those without. Similarly, for patients with HPV- OPSCC, the risk of recurrence in patients with distant metastasis was 4.62-fold higher than that in patients without metastasis (Table 5).

**Table 4:** Disease-free survival rate (%) after 1, 3, 5, and 7 years, stratified according to HPV status in patients with oropharyngeal squamous cell carcinoma

	Time after diagnosis			
	1 year	3 years	5 years	7 years
HPV+	96.6	91.1	90.4	90.1
HPV-	98.1	91.7	89.1	85.9

**Table 5:** Multiple Cox regression model for the risk of recurrence in patients with HPV+ and HPV- oropharyngeal squamous cell carcinoma

Variable		HR	HPV+				HPV-		
			95% CI		p value	HR	95% CI		p value
			Lower	Upper			Lower	Upper	
Metastasis									
	No	1.00	-	-	-	1.00	-	-	-
	Yes	11.75	5.58	24.73	<0.001	4.62	1.62	13.15	0.004

## DISCUSSION

The seven-year OS and DFS of patients with OPSCC were higher among those with HPV+ disease. Among such patients, those with N+ tumors had a lower risk of death, whereas the presence of distant metastasis in both HPV+ and HPV- disease led to a higher risk of death. The 7-year DFS was 90.1% among patients with HPV+ OPSCC and lower (85.9%) among those with HPV- disease. Distant metastasis increased the risk of recurrence of patients with HPV+ OPSCC (11.7 fold) more than twice as much as that among patients in the HPV- group (4.62 fold). An advanced clinical stage (stage IV) was associated with a higher risk of recurrence in the HPV+ group than that in HPV- group.

The prevalence of HPV+ in OPSCC cases increased between 2017 and 2019 (from 21.1% to 32.4%) in developed countries in North America and Europe.^(10)^ In our study, the majority of patients with HPV+ OPSCC were between 50 and 69 years old (66.1%), which is consistent with than in previous studies in which an increase in the mean age of patients with HPV+ was observed over time; the median age of such patients increased from 52 to 59 years between 2002 and 2017.^(29,30)^

In previous reports, patients with HPV+ OPSCC responded better to oncological treatment, with better 2- and 5-year prognoses and OS than those with HPV- OPSCC.^(23,31)^ The 3-year OS rate was 82.4% for HPV+ cases and 57.1% for HPV- cases, depending on the treatment modality.^(32)^ According to Abrahão et al.,^(26)^ patients with HPV- OPSCC have a 3.4-fold higher mortality rate than those with HPV+ OPSCC, and older age is associated with a lower OS. In our study, older age was not a predictive factor for worse survival, regardless of the HPV status.

The most common subsites for HPV+ tumors in this study were the tonsils and the base of the tongue. A systematic review revealed that the prevalence of HPV infection among patients with OPSCC in the tonsils and tongue was higher than that of HPV- tumors, ranging from 56-70%.^(22)^ The impact of HPV infection on prognosis differs among anatomical subsites, both in this study and in previous studies. In this study, the prognosis seemed more favorable for lymphoepithelial tumors of the oropharynx, such as those in the tonsils and base of the tongue, although this result was not significant. This is in contrast to the risk conferred by carcinomas originating in non-lymphoepithelial subsites of the oropharynx, such as the soft palate, uvula, and posterior pharyngeal wall.^(20,23)^ In this study, tumors of the soft palate, a non-lymphoepithelial region, seemed to confer a higher risk of death to patients with OPSCC (again, not statistically significant). The explanation for this apparent prognostic difference remains unclear; however, the presence of lymphoid tissue in the palatine and lingual tonsils may impart a distinct immune functionality, leading to a unique interaction between HPV and the immune system in those sites.^(21,22)^

Smokers with HPV+ OPSCC had a higher risk of death than non-smokers in this study. According to Wilkins et al., patients with HPV+ OPSCC with tobacco exposure exhibit a poorer prognosis than those without those exposure, potentially attributable to smoking-related comorbidities or social determinants of health rather than its capacity to affect tumor recurrence rates.^(33)^ D'Souza et al. reported no significant difference in survival rates among patients with HPV+ OPSCC, regardless of whether they were non-smokers, smokers with <20 pack-years, or smokers with >20 pack-years.^(17)^ Lai et al. noted that HPV+ OPSCC is often associated with alcohol consumption, which diminishes the favorable prognostic effect of HPV positivity.^(34)^ Patients with HPV+ OPSCC who are drinkers reportedly have a twofold higher risk of death than non-drinkers.^(26)^ In our study, former drinkers among patients with HPV+ OPSCC also had a higher risk of death in the univariate analysis but not in the multivariable analysis. Moreover, current drinkers did not have a higher risk.

Individuals diagnosed with clinical stage IV OPSCC had a higher risk of death than those with stage I/II disease in univariate analyses but not in the multivariable analysis; those with stage III disease had a maintained higher risk of death. Kowalski et al. reported a 3.23-fold higher risk of death in patients with stages III/IV OPSCC than those with stages I/II.^(35)^ Lifsics et al. observed a 34% reduction in the risk of early death for patients with an N0 status compared with those with an N+ status.^(36)^ In our study, patients with HPV- OPSCC and metastasis had a 4.62 times higher risk of death than those without metastasis. We could not find previous studies in which the risk of death of patients with OPSCC was stratified according to HPV infection status. However, one study indicated that HPV positivity in OPSCC is characterized by better survival, even in patients with metastasis, owing to a better treatment response.^(37)^

Disease recurrence was higher among patients with HPV+ OPSCC by the 3^rd^ year compared with those with HPV- disease, consistent with the results of Lai et al.^(34)^ In previous studies, patients with HPV+ OPSCC who survived longer than two years seemed to have a lower probability of distant recurrence than those who survived longer (34.6% *versus* 55.0%, p=0.09), and a larger proportion of those who survived longer had multiple recurrence episodes (45.7% *versus* 25.0%, p=0.087).^(27,28)^ However, no consensus is available on whether HPV infection status is associated with recurrence time.^(37-39)^

Guo et al. discovered that patients with HPV+ OPSCC had later disease recurrence than those with HPV- disease, both locoregionally (median of 20.9 *versus* 9.7 months, p=0.069) and metastatically (18 *versus* 11.2 months, p=0.0026).^(28)^ Our study revealed an increase in recurrences 3 years after treatment in patients with HPV- OPSCC, whereas those with HPV+ OPSCC experienced recurrence within 3 years of diagnosis, on average. In other studies, 10%-20% of patients with HPV+ OPSCC developed recurrent disease within 5 years;^(27,28)^ in our study, most OPSCC recurrences also occurred within this timeframe. Our 7-year survival analysis revealed that patients with HPV+ OPSCC had no significant increase in the probability of disease recurrence after the 5^th^ year. This result contrasts the previously reported association of HPV+ OPSCC with later recurrences and the better survival rates reported in studies with shorter follow-up periods.^(28)^

Zhang et al. reported that patients with HPV- OPSCC had a higher risk of death from cancer and other causes than those with HPV+ OPSCC over 5 years (cancer-related deaths: HPV- = 26.9% and HPV+ = 10.7%; death from other causes: HPV- = 13.7% and HPV+ =5.8%). However, our study revealed a homogeneous distribution of cancer-specific deaths and death from other causes between these two groups, suggesting the involvement of other factors, such as age, smoking status, comorbidities, treatment, and the presence of more than one primary tumor.^(40)^ In our study, patients with HPV- OPSCC had higher risks of all-cause and cancer-related death in the first 3 years than patients with HPV+ disease. The OS rate of patients with HPV- OPSCC decreased by 18% between the 1^st^ and 3^rd^ year. Non-cancer-related deaths were more homogeneously distributed regardless of HPV status, which aligns with previous studies that revealed the influence of other risk factors, such as smoking and comorbidities.^(40-42)^ We found no other studies in which the 7-year survival of patients with HPV+ and HPV- OPSCC was reported, stratified according to cancer-specific deaths and deaths from other causes.

One limitation of our study is that HPV infection status was based exclusively on p16 IHC. Mehanna et al. have shown that discrepancies can occur between p16 testing and HPV DNA detection. Discordant profiles (p16-/HPV+ or p16+/HPV-) have intermediate prognoses between those of p16+/HPV+ and p16-/HPV- groups, indicating that p16 alone may not be sufficient for accurate risk-of-death stratification.^(43)^

## CONCLUSION

HPV infection is a significant prognostic factor for overall survival and disease-free survival among patients with oropharyngeal squamous cell carcinoma. In our study, survival analysis exceeding 5 years identified higher overall survival rates in patients with HPV+ oropharyngeal squamous cell carcinoma. Although higher recurrence rates were observed in the initial 3 years, patients with HPV+ disease exhibited lower recurrence rates after the 5^th^ year compared with patients with HPV- disease. Routine p16 IHC testing in therapeutic planning allows the identification of a cohort at risk for both early and late recurrence. We recommend more frequent follow-ups, especially beyond the 5-year mark, for patients with HPV- oropharyngeal squamous cell carcinoma. Metastatic disease is a risk factor for recurrence and mortality in patients with oropharyngeal squamous cell carcinoma. Lifestyle habits such as tobacco and alcohol consumption negatively affect the survival of patients with HPV+ oropharyngeal squamous cell carcinoma.

## SUPPLEMENTARY MATERIAL

### Overall and disease-free survival in patients with HPV-positive and HPV-negative oropharyngeal cancer

Matheus de Abreu, Dandara Menezes de Araujo Oliveira, Bartolomeu Conceição Bastos Neto, Janaina Naiara Germano, Luiz Paulo Kowalski, Maria Paula Curado

**DOI: 10.31744/einstein_journal/2025AO1525**

In the univariate Cox regression analysis of HPV+ OPSCC, the risk of death was higher for individuals with lower educational levels, with a 45% increased risk of death for those with 0-8 years of education compared with those with >11 years of education. Patients with stage IV disease had a 4.54-fold higher risk of death than those with early-stage (I/II) disease. Patients with HPV+ OPSCC T3/T4 had a 2.14-fold higher risk of death than those with T1/T4, and those with distant metastasis had a 3.63-fold higher risk than those without. Patients with OPSCC and more than two primary tumors had a 3.46-fold higher risk of death than those with only one primary tumor. Smokers with HPV+ OPSCC had a 4.74-fold higher risk of death than non-smokers, while former drinkers had a 3.23-fold higher risk than non-drinkers (Table 1S).

**Table 1S:** Univariate analysis of prognostic factors for mortality risk in patients with HPV+ oropharyngeal squamous cell carcinoma

Variable		HR	95% CI		p value
			Lower	Upper	
Sex					
	Female	1.00	-	-	-
	Male	1.39	0.72	2.69	0.314
Education					
	> 11 years	1.00	-	-	-
	0 – 8 years	3.56	1.45	8.73	0.005*
	9 – 11 years	3.91	1.76	8.68	0.001*
Topography					
	Base of tongue	1.00	-	-	-
	Soft palate	1.62	0.48	5.46	0.431
	Tonsils	0.68	0.38	1.22	0.205
	Oropharynx	0.88	0.45	1.72	0.885
Clinical stage					
	I/II	1.00	-	-	-
	III	2.70	1.55	4.70	<0.001*
	IV	4.54	2.27	9.08	<0.001*
TNM classification (T)					
	T1/T2	1.00	-	-	-
	T3/T4	2.14	1.26	3.61	0.004*
TNM classification (N)					
	N0	1.00	-	-	-
	N+	0.52	0.30	0.91	0.024*
TNM classification (M)					
	No	1.00	-	-	-
	Yes	3.63	1.97	6.71	<0.001*
Second primary tumor					
	0	1.00	-	-	-
	1	1.81	0.92	3.58	0.084
	> 1	3.46	1.24	9.61	0.017*
Smoking status					
	Non-smoker	1.00	-	-	-
	Smoker	4.74	2.48	9.06	<0.001*
	Former smoker	2.43	1.25	4.73	0.009*
Alcohol consumption					
	Non-drinker	1.00	-	-	-
	Drinker	1.37	0.76	2.34	0.311
	Former drinker	3.21	1.63	6.31	0.001*

* Statistically significant (p<0.05).

In the simple Cox regression analysis of HPV- OPSCC, individuals with lower educational levels exhibited a higher risk of mortality, with a 3.34-fold increase in mortality risk for those with 0-8 years of schooling compared with those with >11 years of schooling. Patients with clinical stage IV disease had a 2.27-fold higher risk of mortality than those with stage I/II disease. HPV+ OPSCC T3/T4 tumors were associated with a twofold higher risk of mortality compared with T1/T2 tumors. Individuals with distant metastases had a 4.8-fold higher risk of mortality than those without, whereas those with more than two primary tumor had a 60% (but non-significant) lower risk of mortality than those with only one primary tumor (Table 2S).

**Table 2S:** Univariate analysis of prognostic factors for mortality risk in patients diagnosed with HPV- oropharyngeal squamous cell carcinoma

Variable		HR	95% CI		p value
			Lower	Upper	
Sex					
	Female	1.00	-	-	-
	Male	0.76	0.40	1.43	0.403
Age					
	18-49	1.00	-	-	-
	50-69	0.67	0.28	1.59	0.374
	70+	0.70	0.27	1.78	0.459
Race/ethnicity					
	White	1.00	-	-	-
	Black/brown	1.10	0.60	2.02	0.741
Education					
	> 11 years	1.00	-	-	-
	0 – 8 years	3.34	1.23	9.10	0.018*
	9 – 11 years	2.66	0.81	8.73	0.105
Anatomical site					
	Base of tongue	1.00	-	-	-
	Soft palate	0.46	0.20	1.05	0.068
	Tonsils	0.88	0.47	1.65	0.701
	Oropharynx	0.97	0.50	1.85	0.933
Clinical stage					
	I/II	1.00	-	-	-
	III	1.56	0.65	3.73	0.315
	IV	2.27	1.06	4.87	0.034*
TNM classificaiton (T)					
	T1/T2	1.00	-	-	-
	T3/T4	2.00	1.12	3.56	0.018*
TNM classificaiton (N)					
	N0	1.00	-	-	-
	N+	1.12	0.66	1.91	0.663
TNM classificaiton (M)					
	No	1.00	-	-	-
	Yes	4.80	2.58	8.94	<0.001*
Second primary tumor					
	0	1.00	-	-	-
	1	0.70	0.34	1.44	0.342
	> 1	0.40	0.09	1.67	0.212
Smoking status					
	Non-smoker	1.00	-	-	-
	Smoker	1.27	0.60	2.68	0.518
	Former smoker	1.43	0.66	3.09	0.356
Alcohol consumption					
	Non-drinker	1.00	-	-	-
	Drinker	0.80	0.41	1.54	0.508
	Former drinker	0.96	0.48	1.90	0.906

* Statistically significant (p<0.05).

In the simple Cox regression analysis of HPV+ OPSCC, the risk of recurrence was significantly associated with clinical staging; patients with stage IV disease had a 5.96-fold higher risk of recurrence than those with stage I/II disease. Individuals with distant metastases had a more than 10-fold higher risk of recurrence than those without. Alcohol users and patients with N+ tumors seemed to have an increased recurrence risk, although these results were not significant (Table 3S).

**Table 3S:** Univariate analysis of prognostic factors for recurrence risk in patients with HPV+ oropharyngeal squamous cell carcinoma

Variable		HR	95% CI		p value
			Lower	Upper	
Sex					
	Female	1.00	-	-	-
	Male	1.28	0.49	3.36	0.611
Age					
	18-49	1.00	-	-	-
	50-69	0.59	0.21	1.62	0.314
	70+	0.94	0.30	2.88	0.917
Race/ethnicity					
	White	1.00	-	-	-
	Black/brown	1.50	0.64	3.51	0.348
Education					
	> 11 years	1.00	-	-	
	0 – 8 years	1.14	0.24	5.29	0.863
	9 – 11 years	1.81	0.64	5.10	0.257
Anatomical site					
	Base of tongue	1.00	-	-	-
	Soft palate	2.21	0.25	18.91	0.469
	Tonsils	1.92	0.70	5.20	0.200
	Oropharynx	1.38	0.42	4.52	0.595
Clinical stage					
	I/II	1.00	-	-	-
	III	1.17	0.44	3.09	0.740
	IV	5.96	2.57	13.79	<0.001*
TNM classification (T)					
	T1/T2	1.00	-	-	-
	T3/T4	1.27	0.60	2.60	0.539
TNM classification (N)					
	N0	1.00	-	-	-
	N+	3.08	0.73	12.99	0.124
TNM classification (M)					
	No	1.00	-	-	-
	Yes	10.22	4.86	21.48	<0.001*
Second primary tumor					
	No	1.00	-	-	-
	Yes	2.03	0.82	5.00	0.121
Smoking status					
	Non-smoker	1.00	-	-	-
	Smoker	1.45	0.58	3.61	0.420
	Former smoker	1.25	0.53	2.96	0.598
Alcohol consumption					
	Non-drinker	1.00	-	-	-
	Drinker	1.69	0.77	3.69	0.186
	Former drinker	0.83	0.18	3.80	0.814

* Statistically significant (p<0.05).

In the simple Cox regression analysis of HPV- OPSCC, the risk of recurrence was 4.05-fold higher for individuals with distant metastasis than for those without. Self-reported black or mixed-race patients seemed to have a higher risk of recurrence than self-reported white patients, although this was not significant (Table 4S).

**Table 4S:** Univariate analysis of prognostic factors for recurrence risk in patients with HPV- oropharyngeal squamous cell carcinoma

Variable		HR	95% CI		p value
			Lower	Upper	
Age					
	18-49	1.00	-	-	-
	50-69	1.79	0.23	13.60	0.570
	70+	1.69	0.20	14.08	0.625
Race/ethnicity					
	White	1.00	-	-	-
	Black/brown	1.78	0.69	4.59	0.232
Education					
	> 11 years	1.00	-	-	-
	0 – 8 years	1.29	0.41	4.07	0.662
	9 – 11 years	1.41	0.33	5.93	0.632
Anatomical site					
	Base of tongue	1.00	-	-	-
	Soft palate	1.44	0.41	4.98	0.562
	Tonsils	1.20	0.36	3.94	0.759
	Oropharynx	1.43	0.43	4.69	0.552
Clinical stage					
	I/II	1.00	-	-	-
	III	1.51	0.38	6.07	0.555
	IV	1.64	0.46	5.77	0.436
TNM classification (T)					
	T1/T2	1.00	-	-	-
	T3/T4	1.27	0.51	3.16	0.597
TNM classification (N)					
	N0	1.00	-	-	-
	N+	1.27	0.51	3.15	0.604
TNM classification (M)					
	No	1.00	-	-	-
	Yes	4.05	1.57	10.47	0.004*
Second primary tumor					
	No SPT	1.00	-	-	-
	With SPT	0.94	0.34	2.56	0.912
Smoking status					
	Non-smoker	1.00	-	-	-
	Smoker	0.87	0.23	3.29	0.844
	Former drinker	1.67	0.46	5.99	0.430
Alcohol consumption					
	Non-drinker	1.00	-	-	-
	Drinker	0.84	0.26	2.75	0.785
	Former drinker	1.22	0.37	3.97	0.738

* Statistically significant (p<0.05).
